# Chinese equestrian policy development: a narrative review

**DOI:** 10.3389/fvets.2023.1281019

**Published:** 2024-01-04

**Authors:** Jiaxin Li, Raúl Sánchez-García

**Affiliations:** Facultad de Ciencias de la Actividad Física y del Deporte (INEF), Universidad Politécnica de Madrid, Madrid, Spain

**Keywords:** policy, equine, development issues, history, sport management

## Abstract

**Introduction:**

This paper provides an overview of the Chinese equestrian policy documents and regulations from 1978 to 2022. While the horse business is shifting from traditional to leisure and sport pattern in China, through the analysis of the policies and regulations.

**Objectives:**

This paper aims to provide a concise overview of the government's policies which growth of equestrian sports in China over the past four decades (since 1978).

**Method:**

Under the guidance of Whitemore and Knafl's 5-step approach to policy analysis, a systematic analysis of policy content, context, and governance processes is conducted. As of 2022, 29 documents from official and semi-official sources had been extracted, classified, and examined for records.

**Results and conclusion:**

As of 2022, 29 documents from official and semi-official sources had been extracted, classified, and examined for records. The first is whether the policy is useful or not, its goals are vague and unclear. And there isn't much interaction between the areas it affects. Second, the policies are inconsistent and unstable. Third, there is a deficiency in terms of professional evaluation and relevancy. Fourth, pre-development preparation is not done due to the lack of an industry chain cycle. The complex causes of these issues include the sports management system, the government's policy ideals, and the competition of interests among policy stakeholders.

## Introduction

In the eighteenth century, a modern form of equestrianism was created in the world (1). Later in 1900, equestrianism became an official event at the modern Olympic Games (2). For the 1912 Stockholm Olympics, equestrian events returned to the Olympics after being absent for a short time (3). In 1952, female riders began to be allowed to participate in the Olympic Games, and equestrians became the only competition in the Olympic Games where men and women, no matter how old riders were, could all compete on the same field (4). Until now, equestrian has been the only event in the Olympics where humans and animals in a team compete simultaneously.

In Chinese history, horses played an important part. Equestrianism in China can be traced back to the Zhou Dynasty (1046 BC - 256 BC). In the Tang dynasty (AD 618 - 918), horse Mingqi^1^ showed the position and rank of their forms and scale (6). At the beginning of the twentieth century, horse racing and polo were brought to Shanghai and Hong Kong. This brought back interest in horses' value potential in China.

The Chinese Equestrian Association (CEA) was established in 1979 and joined the FEI in 1983 (7). Since then, the CEA has worked to help equestrian sports grow in China. In 1984, China joined the Olympic Games, but no Chinese equestrians participated until the 2008 Beijing Olympics. It marked the beginning of equestrian development for China, even though it had 300 equestrian clubs when six equestrian athletes entered the Olympic stadium during the 2008 Beijing Olympics (8).

Sports have been recognized as an essential sector in China (9). The Chinese sports business began 40 years ago and is still considered a developing sector. The Chinese government has taken several steps to improve sports. These include putting a lot of money into sports development, encouraging more people to play sports, and putting in place policies and programs to find and train young talent. In 1998, when the Chinese sports business started, the Chinese government established a hierarchical sports system, the General Administration of Sport (GAS), which is responsible for all sports activity. In China, the undertaken of the equestrian sports are related to the Olympic games and traditional cultural competitions, such as ethnical equestrian sports like speed horse racing in Inner Mongolia (10).

Due to the continued growth of the global horse industry, which was led by developed Western countries, and the decline of traditional horse industries (agriculture, transportation, and the military), a new modern horse industry has been created (11). This industry is based on sports, leisure, and product consumption, and it connects the primary, secondary, and tertiary sectors (12). An industrial chain was also made to deal with the change in how horses were used in society. Even though it is less developed than in the Western countries, China's horse industry chain includes: breeding horses, training horses, giving horses shots, horse trading, and making horse equipment. While China's horse industry chain is far from flawless, it is on the road to improvement. The growth of the Western horse industry can teach China a lot about how to build equestrian and horse businesses (13).

Compared to the 1977 horse stock, when China's horse stock was the world's largest at 11.45 million, the national horse stock reached 3.671 million in 2019, accounting for 6% of the world's total stock and ranking fifth (14). But now, compared with other countries like Germany, China does not have the same advantages as before. German has led to the growth of a sizeable equestrian industry, which has created 300,000 jobs (15). This starkly contrasts with China's current equestrian industry.

China's horse industry has moved into a new phase. The government has seen that horseback riding could benefit the economy, especially regarding tourism and the export of horses to Europe. China's governments realized the new development concept of building and systematically promoting “human-centered, human-horse integration” in equestrianism (16). Because of this, the government has helped the sport grow by funding training centers and giving tax breaks to businesses in the equestrian industry chain (17).

There are also some challenges. Many individuals, especially those in rural regions, lack access to horses and training facilities. The high expense of engaging in equestrian activities is another obstacle restricting the sport's accessibility. There are also animal welfare issues, notably around the usage of horses in sports.

This paper aims to comprehensively explore the evolution of China's equestrianism “policy,” highlight current challenges, and provide insightful commentary in the discussion section. The structure of the paper is as follows: the first section provides a concise overview of the existing literature on Chinese sports policy; the second section outline the methods used for the analysis; the third presents the development of equestrianism policy and regulation, which categorized into three phases: the budding phase (1978–2004), the preparation phase (2005–2012), and the development phase (2013–2022). Finally, the discussion section critically assesses the findings, pinpointing key problems and challenges of Chinese equestrianism.

## Chinese sports system review

**Figure 1:**
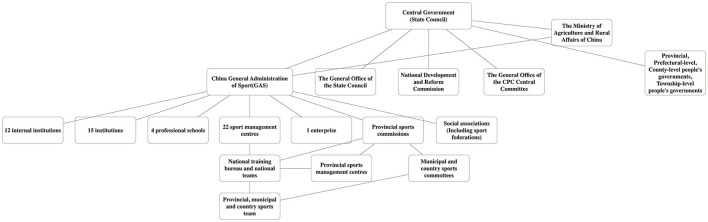
Sports system in China.

China's sports policy in various areas hasn't evolved in line with recent changes and trends. Li et al. focused primarily on the recent evolution of equestrianism in China (18). We used Figure 1 to make a simple explanation.

Despite the invaluable insights from past studies, there hasn't been a systematic examination of the evolution of China's equestrian policies over time. This paper aims to address this gap by analyzing primary documentary sources.

## Methods

This study employs a qualitative analysis of Whitemore and Knafl's 5-step method of China's equestrian policies and regulations documents spanning from 1978 to 2022 (19). When policies are viewed as “a collection of processes that occur in sequence” which “reflect past, present, and future discussions,” policy papers can be perceived as “a specific point in the materialization of the policy process intended to represent or enact power and change (20).” China has a consistent history of “documentary politics,” meaning the use of policy documents to structure national matters (21). Policy documents, which include decisions, directives, opinions, rules, notices, and explanations issued by the National People's Congress, the Central Committee of the Communist Party of China (CPC), the State Council, and other state organizations, carry legal weight (22). These policies might signify the central government's overarching policy goals or specify governance mechanisms (23).

The equestrian industry policy encompasses a set of policies introduced by federal, provincial, and municipal governments to actively engage in the equestrian industry and associated economic activities. These regulations aim to manage the equestrian market's expansion, allocate resources efficiently, foster the growth of the equestrian industry, and expediently transition China's conventional horse industry into a contemporary equestrian sector. Given the unique nature of equestrian sports, they cannot be isolated from the broader equestrian industry. Equestrian athletes need training, and horses necessitate a market. Without a market, horse breeding promotion becomes challenging, and the assurance of producing high-quality, competitive horses dwindles. These aspects encompass a myriad of elements and touch on countless facets of decision-making, debate, and evolution in daily sporting activities, making their presence ubiquitous (24).

More specifically, our methodology involves qualitative documentary analysis. Four primary sources contributed to our material compilation: (1) Official websites and internal documents from the GAS of China, The Ministry of Agriculture and Rural Affairs of China, provincial sports policies, and Chinese sports federations. We also sourced relevant content from the China Hownet, Web of Science, and EBSCO databases; (2) Pertinent papers from Chinese administrative and commercial entities; (3) Insights into the ramifications of sports industry policies, gleaned from Chinese business unit websites; and (4) semi-official resources (from media and organizations) such as Sina News and Equestrian Online. Table 1 delineates these data sources.

**Table 1:** The research sources detail.

**Category**	**Resources**
Government	The Government of Xinjiang Uygur Autonomous Region of China The Government of Inner Mongolia Autonomous Region of China Department of Agriculture and Animal Husbandry of Inner Mongolia Autonomous Region of China The General Office of the CPC Central Committee and the General Office of the State Council Hulunbuir Municipal Government The Ministry of Agriculture and Rural Affairs of China, General Administration of Sport General Administration of Sport Jimnai County People's Government General Office of the State Council
Sports association	China equestrian association
Academic	China Hownet Web of Science EBSCO databases
	Related publications, including policies, sports, and history
Semi-official media	News media Organization
	Souhu news; Sina news Equestrian Online (25)

All policy documents underwent a two-step selection procedure. Initially, we utilized search engines and databases to probe for terms like “马术/equestrian sports,” “马产业/horse industry,” and “马/horse” within the aforementioned resources. This search was complemented by a literature review on equestrianism and China's horse industry to unearth additional relevant policies. We then filtered out content unrelated to equestrian sports and horse industry policy, such as articles on horse science and equine medicine. All chosen documents span from 1978 to 2022.

**Figure 2:**
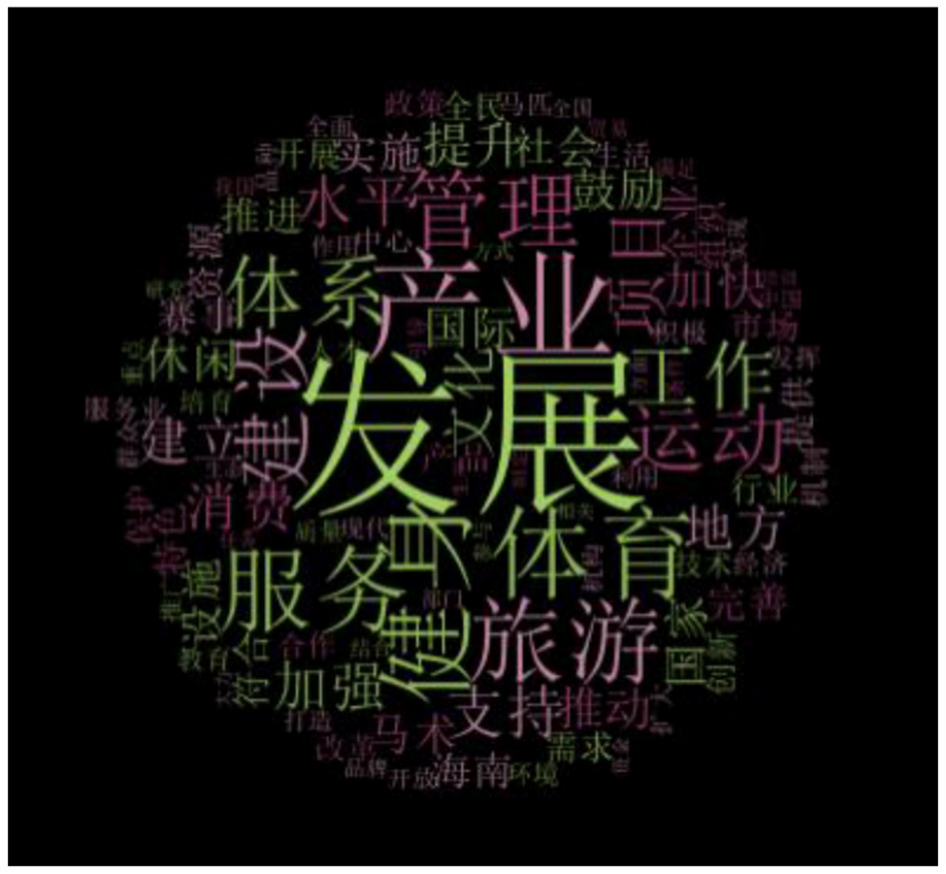
Nvivo policies word frequency.

For analysis, we employed the Nvivo 20 software on all full-text documents in Chinese. Our approach began with open coding, succeeded by clustering and categorization. Using Nvivo's word frequency tool, we categorized the primary verbs in each document, as illustrated in Figure 2, presented in Chinese. Initial coding helped segregate the documents into three distinct epochs. We then chronologically arranged the data and grouped it into three pivotal horse-related policy development phases based on timeline milestones. Following this, we embarked on a categorization phase, structuring thematic concepts rooted in relevance to comprehensively grasp the nuances of China's policy during various stages. This information was synthesized to produce an interpretive overview that encapsulated the findings of our study.

All original documents were in Chinese, and the team handled translations into English. English references from government websites, press releases, and scholarly articles were utilized to authenticate these translations. For certain terms, Chinese pinyin was favored over English translations to better convey indigenous concepts.

## Chinese equestrian policies development process

Policy trajectory analysis shows how equestrian sports and horse-related businesses have grown in China over the last 50 years. Changes take place in three phases: during the budding phase (1978–2004), Chinese policy mainly focused on horse industry research, transitioning from “establishments” to “implementation of plans;” the preparation phase (2005–2012) implied a shift from actively “joining” international equestrian competitions to “hosting” international equestrian competitions, attracting more riders to compete in China, strengthening exchanges among riders, promoting technical improvement, and club development; the development phase (2013–2022) involved developing policies that would “promote” and “plan” for better growth in the future. Table 2 provides an overview of the Chinese equestrianism development process, including the critical policies introduced at each stage and the timeline.

**Table 2:** Evolution of equestrianism development in China.

**Stage**	**Date**	**Event**	**Main contents**
Initial phase (1978–2004)	1978	Third Plenary Session of the 11th Central Committee of the Communist Party of China held in 1978	Sports socialization became the primary purpose of sports development policy.
	1979	Chinese Equestrian Association was established	The start of the transition to modern horse competition.
	1982	Join FEI	Officially became a member of the FEI.
	1986	Sports lottery (26)	Authorization
	1992	Notice on Resolutely Stopping Horse Racing Betting and Other Activities of Gambling Nature	Stop Horse Racing
	1999	Several Opinions on Promoting the Construction and Development of Hainan International Tourism Island	Stop Horse Racing
Preparation phase (2005–2012)	2005	Chinese judge in FEI	Qualification to register with the FEI.
	2008	Beijing Olympic Games	The Chinese equestrian team competed in the Olympics for the first time.
	2010	The first executive meeting to consider the adoption, effective 1 May 2010	Animal epidemic prevention conditions.
	2011	The Bird's Nest hosted the International Equestrian Masters event	The first time a commercial equestrian event entered China
	2012	Held the first junior equestrian championship	
Development phase (2013–2022)	2014	N0.46 expanded the sports industry market (27)	2025 sports industry program objectives.
	2015	No. 85 (28)	Expand market-oriented service supply service industry and sports industry.
	2016	National Fitness Plan (2016–2020) (29)	2020 national fitness objectives.
	2016	No. 77 (30)	Solves the problem of sports occupation in No. 46.
	2016	Guiding Opinions of the General Office of the People's Government of Xinjiang Uygur Autonomous Region on Accelerating the Development of the Modern Horse Industry (31)	Providing horse related employment opportunities.
	2017	Several Opinions of the People's Government of Inner Mongolia Autonomous Region on Promoting the Development of Modern Horse Industry (32)	Preserving and improving horse breeding.
	2018	Implementation Program of Key Projects for the Development of Modern Horse Industry (33)	Investment in equine development.
	2018	Guiding Opinions on Supporting Hainan's Comprehensively Deepening Reform and Opening-up (34)	Urge Hainan to promote horse racing sports and encourage the study of competitive sports lotteries and significant international event lotteries.
	2018	Implementation Plan for the Construction of an International Tourism Consumption Center in Hainan Province (35)	Promote the evolution of horse racing.
	2019	Outline Development Plan for the Guangdong-Hong Kong-Macao Greater Bay Area (36)	Support the development of equestrian industry.
	2020	Hulunbuir City Modern Horse Industry Development Plan (2019–2025) (37)	Support the development of equestrian industry.
	2020	National Horse Industry Development Plan (2020–2025) (38)	2025 Plan for the Equine Industry to Achieve Relevant Targets
	2021	National Fitness Plan (2021–2025) (39)	Development of sports
	2021	Development Plan of Modern Horse Industry in Altai Region (40)	Support the development of equestrian industry.
	2021	Letter from the State Office [2021] No. 79 (41)	In 2025, Hong Kong, Macau, and Guangdong will jointly organize the 15th National Games.
	2021	Measures for the Administration of Registration and Training Assessment of Equestrian Coaches in China (42)	Classification of Coaches' Levels.
	2021	“Letter on the Reply to the Proposal No. 3627 (No. 326 for Medical and Physical Education) of the Fourth Session of the Thirteenth National Committee of the Chinese People's Political Consultative Conference_State Sports General Administration” (17)	Suggestions on establishing a Chinese equestrian system; promoting the balanced development of equestrian events and further promoting horse training events; equestrians into campus; strengthening international exchanges and introducing professional talents; clarifying the land used for equestrian sports and standardizing the approval process
	2022	China Equestrian Association Rider Grading Rulebook (43)	Specific requirements for graded testing of riders' ratings.

### The budding phase from “establish” to “implement:” 1978–2004

During this time, all directions for development were uncertain, and some projects would be stopped if they did not work out as planned. Horse racing was one of these projects to be discussed and “implemented” (44). According to Nvivo's policy classification, the most important keywords in this budding stage were “established,” “implemented,” “unplanned,” “stop,” and “try” (45). As no appropriate growth plan was 'established,' we defined this period as the first budding phase.

At the Third Plenary Session of the 11th Central Committee of the Communist Party of China, held in 1978, it was clear that the socialization of sports became the primary purpose of sports development policy (46). The CEA was “established” in 1979, which we regarded as the beginning of the equestrian industry. This also indicated that Team China was preparing to join equestrian competitions.

Horse racing had been a part of the horse business since ancient China (47). With political supervision and pressing economic benefits, commercial horse racing was carried out in Shenzhen, Guangzhou, and other coastal cities. However, there were many problems, and the relevant government departments tried to stop it several times (48). Until 2002, five ministries and commissions, including the Ministry of Public Security, the Ministry of Supervision, the State Administration for Industry and Commerce, the GAS, and the National Tourism Administration, jointly issued a document stating that the operation of horse racing activities with gambling was strictly prohibited (49). In the middle of the nineteenth century, Chinese sociologists, historians, and folklorists studied traditional Chinese horse racing (50). Yet, no researcher paid attention to the sports aspect or considered how to continue using the facilities to support the horse business. The equestrian business was neither investigated nor taken seriously at that time.

Authorizing the sports lottery was “implemented” in China's sports history in 1986 (51). However, horse racing was not mentioned since it was not legally permitted in China. Administrative methods no longer governed horse racing. According to unofficial statistics (from website news), China's sports sector generated 1.6 billion RMB in income from 1978 to 1992. The money generated by the sports system accounted for less than one-tenth of the total sports input in 1992 (52).

At that time, there was no precise definition of the sports industry. Also, China's equestrian industry was mainly “unplanned.” The origin of the modern equestrian industry in mainland China can be traced back to the early 1990's when the state allowed some economically developed regions to “try” and set the modern equestrian industry (53). In those days, contemporary equestrianism was mainly a spectator sport, and equestrian show jumping was primarily an entertainment and performance show.

Overall, there was no coordinated application of horse industry policies during this period. As a result, the equestrian industry stagnated.

### Preparation phase-“join,” “hold,” and “first:” 2005–2012

After establishing a development direction in the first phase, the objectives became clearer as equestrian and other horse-related industries developed. The keywords for this stage are “join,” “hold,” and “first.” The direct testimony lays a solid foundation for future development; therefore, we consider this stage a preparation phase. Governments planned to introduce associated policies to improve it gradually. The key achievement was that the “first” Chinese judges qualified to “join” the FEI in 2005. This helped establish regulation and teaching methods in equestrianism, laying the foundation for the future development of China's equestrian industry.

The 2008 Beijing Olympic Games marked a significant milestone in elevating equestrianism to a new level. During these Games, China had the opportunity to showcase itself and, for the “first” time, Team China “joined” equestrian competitions. At that time, the location of the equestrian competition was moved to Hong Kong, prompting many discussions (54). Hong Kong had better conditions and more experience organizing international horse events. In contrast, Beijing lacked the necessary facilities and experience. After much deliberation, it was decided on 8 July 2005 that Hong Kong would co-host the 29th Beijing Olympic Equestrian Events.

One notable event in 2008 was the change of nationality of Huatian Alex to Chinese. Huatain is the first rider to represent China in the Olympic equestrian eventing event (55). This marked the “first” time the Chinese equestrian team competed in the Olympic Games. In total, six riders competed in dressage, showjumping, and eventing. Of the six Chinese riders who “joined” the Olympics, only Hua Tian ranked 25th in the FEI, highlighting a considerable gap between Chinese equestrians and the international level (56).

**Figure 3:**
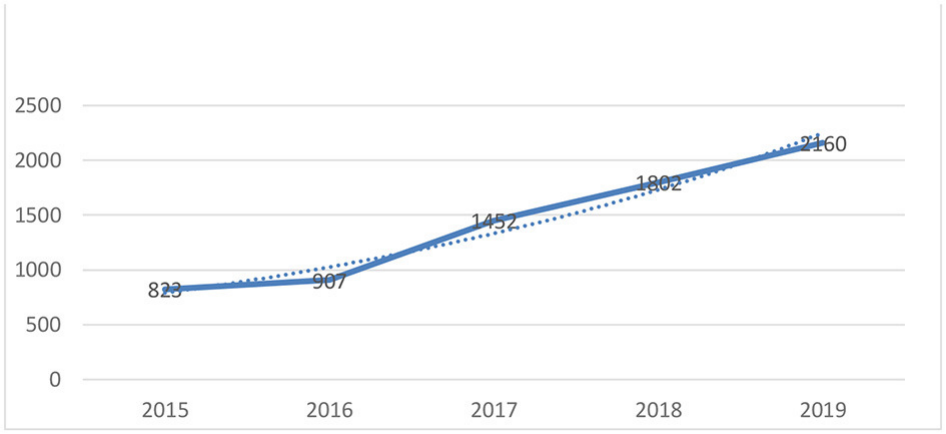
Equestrian clubs in China.

The 2008 Beijing Olympics boosted the sports industry, with many individuals taking interest and “joining” equestrian sports. After the Games, show jumping, dressage, and eventing saw better development in China, as they were listed in the Olympic program. The Olympic Games played a pivotal role in promoting the development of equestrian sports in China. In 2008, there were fewer than 100 horse clubs in China. However, from 2015, the number increased dramatically from 823 to 2,160 [see Figure 3 (18)].

With the development of the equestrian sport, some essential documents about horses were also issued. According to the Ministry of Agriculture, “the first executive meeting to consider the adoption, effective 1 May 2010,” was held on 4 January 2010 (57). This provides the “first” adequate logistical and political support for developing equestrian sports and clarifies the inspection criteria for companies that operate horse activity areas.

After the 2008 Beijing Olympic Games, the Bird's Nest “held” the International Equestrian Masters event for the “first” time in 2011 (58). This indicated that high-level international equestrian events for Chinese riders would be introduced, allowing more riders to “join,” compete, and interact with international riders. In 2012, China “held” its first junior equestrian championship, spurring an equestrian trend among youth and children. With an increasing number of equestrian show jumping competitions for various levels and age groups since 2008, including the introduction of world-level 2-star to 5-star events in China and youth events, the equestrian industry market began to boom.

### Development phase “plan,” “improve,” and “promote:” 2013–2022

In this development phase, the attributes of China's equestrian industry became clear and well-defined, and the country officially entered the development phase. The policy's keyword phrases were “plan,” “improve,” and “promote.” The primary focus of the policy was on rapid development. Several equestrian industry-related policies were released. Objectively speaking, these policies, to a certain extent, fostered the healthy development of the equestrian industry and laid the foundation for its subsequent growth. The equestrian industry policies at this stage could be divided into the following categories: approaches that establish the positioning of equestrianism; guidelines for developing equestrianism in relation to the reform of Chinese sports; and policies concerning equestrianism or other related horse fields.

The release of Document “No. 46” in October 2014 was a significant step forward for the growth of the equestrian industry (59). The primary goal of Document “No. 46” was to support the growth of China's sports industry. The 2015 State Council Document No. 85, “Guiding Opinions on Accelerating the Development of the Consumer Service Industry and Promoting the Upgrading of Consumption Structure,” provided further instructions for developing the sports service industry and promoting the upgrade of the consumption structure (28). The release of “No. 46” set off a surge in investment in China's sports industry, which was already on the rise. The most notable aspect of this document was its alignment with national strategy. However, some of the challenges outlined in “No. 46” are difficult to address; the primary reason being that the lack of sports venues is an issue that spans a scope beyond the jurisdiction of the sports sector.

In 2015, the “Central Committee of the Communist Party of China on the formulation of the thirteenth 5-year plan for national economic and social development” put forward the following statement: “to strengthen the construction of ecological civilization, adhere to green development, form a new pattern of modernization, foster the harmonious development of man and nature, and promote the harmonious coexistence of man and nature (60).” According to this policy, equestrianism would be suitable and in line with these objectives. The utilization of horses in different activities, including equestrian sports and recreational riding, may be regarded as a form of environmentally conscious interaction. For example, horse riding can facilitate the exploration of natural landscapes in a low-impact manner, encourage the participation in outdoor recreation, and cultivate a communion between humans and the environment. Furthermore, equestrianism has the potential to boost rural development and safeguard traditional practices, which is consistent with the overall objectives of the 5-year plan's modernization pattern. More importantly, this approach has the potential to incorporate sustainable practices and traditional cultural elements into the development agenda, thereby contributing to a symbiotic connection between humanity and the environment.

Benefiting from the popularity of sporting events such as the Tokyo Olympics and the Shanxi National Games, equestrianism gradually transitioned from being an aristocratic sport to a more widespread discipline. Consequently, equestrian clubs have proliferated in the country. Horsemanship Magazine China, the leading publication for the equestrian industry in China, indicates that the country has grown from 907 horse farms in 2016 to 2,107 in 2020, with a 5-year growth rate of 57%.

Also on the rise were equestrian enthusiasts, whose numbers surged from about 100,000 to a million in just a few years. Active equestrian members accounted for 52% of the total (61). In 2011, there were only about 10 stores throughout the country, with the industry's annual sales around 5 million RMB. By contrast, in 2017, the number of tack stores had grown to 50, and the annual sales had expanded to 300 million RMB (62). According to the 2017 China Equestrian Market Development Status Report, the average sales of equestrian clubs nationwide reached 6.37 million yuan in 2017 (61). With 1,452 clubs in the country, the total sales of equestrian clubs nationwide amounted to 9.24924 billion RMB in that year (63). The popularity of equestrian sports in China has surged from 2013 to 2022.

Since 2015, policies on developing equestrian sports have been actively formulated from the central to local levels. In 2016, the state council released the “National Fitness Plan (2016–2020),” which designated equestrianism as a sport to be “actively cultivated” (29). That same year, marking the second anniversary of the “No. 46” release, the General Office of the State Council introduced the “Guiding Opinions on Accelerating the Development of the Fitness and Leisure Industry” (“No. 77”), projecting a full scale of 3 trillion RMB in the sports market (30). The goal was to shift sports activities away from urban centers, to prevent congestion on urban land, while “strongly advocating” for the development of high-value industries such as ice and snow sports, outdoor mountain sports, equestrianism, golf, and others (64). Xinjiang, Inner Mongolia, and other provinces and autonomous regions with strong ties to the horse industry also issued specific policies, solidifying the establishment of equestrian clubs, horse breeding farms, and other related industry chains (28, 65).

The Outline of the Guangdong-Hong Kong-Macao Greater Bay Area Development Plan, unveiled in February 2019, emphasized “promoting” the growth of equestrian sports and associated industries. It also sought to enhance cooperation between Hong Kong and the Mainland in the inspection, quarantine, and customs clearance processes for horses, forage, and feed, as well as veterinary drugs, biological products, and other inbound and outbound items (36). Later that year, on August 2nd, the CEA announced the “Measures for the Classification of Equestrian Coaches (Draft for Comments)” and the “Implementation Rules for the Management Measures for Equestrian Coaches (Draft for Comments) (66).”

Due to a lack of relevant regulations in the budding stages, even though equestrianism “developed rapidly,” some equestrian clubs (especially the newly established ones) had to lower their standards because of the difficulty in finding professional coaches. Riders without coaching certificates or young pastoral teenagers were trained briefly before teaching club members how to ride (67).

Human safety concerns also hindered the healthy growth of equestrian sports. It wasn't until August 2019 that the CEA implemented regulations stipulating an age requirement of 18 for equestrian coaches. While the qualifications for equestrian coaches were categorized as A-level, Class B, Class C, Class D, and Class E, there were no specifics about how to obtain these certificates or the assessment criteria for each level (68).

From FEI^2^ (Fédération Équestre Internationale) public database in Table 3, it's evident that Chinese riders have become more prevalent in recent years. A total of 1,175 Chinese riders have already competed. Male riders remain the majority. Furthermore, the number of veterinarians has grown due to FEI initiatives; from 2019 to 2022, the Chinese FEI approved an increase of 18 treating veterinarians.

**Table 3:** FEI official data about Chinese members.

**FEI official group**		**Number**	
Judge		8	
FEI permitted treating veterinarian		18	
Trainer		185	
Owner		262	
Athlete	Female	396	1,175
	Male	908	

^*^Data from Equestrian Magazine (69).

On 29 August 2020, the Ministry of Agriculture and Rural Affairs and the State General Administration of Sports jointly published the “National Horse Industry Development Plan (2020–2025)” on the Ministry of Agriculture and Rural Affairs website (38). The document outlined the present state of China's horse industry, which highlighted that regions are relatively concentrated and the total breeding volume is substantial. It also notes continuous improvements in the breeding system for fine breeds and the growth in the equestrian sports industry. In addition, horse tourism is becoming more prevalent, and international exchanges are intensifying. Additionally, the document identified several challenges that development encounters, including inadequate resources for equine breeds, antiquated production methods, fragile connections in the industrial chain, as well as defective markets.

This was the first “development plan” issued for the horse industry, covering various horse-related sports, including equestrian, polo, and others. It was projected that by 2025, the framework and system for developing China's modern horse industry would be initially established, and the integrated development pattern of the first, second, and third industrial sectors would also begin to take shape. What's more, this document establishes specific objectives: establishing an effective breeding system; enhancing the equestrian activities system; fostering collaborative industry development; and collaborating with educational institutions to cultivate the necessary talent pool for the horse industry.

The plan introduced the “horse industry +” development model, aiming to “promote” the conversion of resource advantages such as horse breeds, equestrian sports, and cultural tourism into economic benefits. It emphasized “increasing” the proportion of the output value of the horse industry and fostering a synergistic development mechanism. Specifically, the importance of equestrian development was highlighted, suggesting that equestrianism is crucial for the evolution of the modern horse industry.

To establish China's equestrian club rating system and “promote” the standardization, uniqueness, and branding of equestrian clubs, there was an emphasis on developing several flagship equestrian clubs. The plan combined equestrian “promotion,” popularization, and “improvement,” leveraging the platform created in preparation for the Olympics to robustly publicize and advance equestrianism. Concurrently, they sought to “promote” the enhancement of the competitive standard of equestrian sports by organizing a series of competitions and training events and hosting various equestrian activities to “boost” the general public's awareness of equestrian sports.

The “Letter of the General Office of the State Council on the Consent of Guangdong, Hong Kong, and Macao to Host the 15th National Games in 2025,” released in September 2021, granted Guangdong, Hong Kong, and Macao the rights to host the 15th National Games in 2025. Intriguingly, this coincided with the year when the Hong Kong Jockey Club's Guangzhou Conghua Racecourse was slated to host a regular event. Institutions of higher education, equestrian clubs, and equestrian events all contributed to the thriving equestrian industry. The CEA established the “Equestrian Big Data Platform” to cater to the equestrian industry chain. Equestrian enthusiasts, riders, and industry professionals could readily access information on the platform concerning equestrian exams, training, and registered events. Details about the horses, event results, any riders injuries, and point rankings were also transparently displayed, making it conducive for horse trading.

The combined effect of several policies and incentives for the equestrian industry played a pivotal role in solidifying the foundation of the equestrian industry, enhancing the standard system, augmenting support, promoting equestrianism, and educating a broader audience about this venerable sport.

## The existing issues and recommendations

As the results have shown, horse-related policies and regulations in China's equestrian sports and horse industry have progressively developed and improved over the past five decades. Nonetheless, some problems still linger in China's Equestrian Industry Policies and Regulations:

First, there's the inadequate interrelatedness of policies. The objectives of the policy were vague and ambiguous, and the sectors they applied to were not interconnected. This led to policies that, while beneficial, were challenging to implement. For instance, the General Office of the Ministry of Agriculture and Rural Affairs and the General Office of the State Sports General Administration introduced the “National Horse Industry Development Plan (2020–2025).” It sets out the framework for the short-term development of China's modern horse industry. The development environment of China's modern horse industry tends to be stable. Although the policies in related fields are scattered, promoting the development of China's modern horse industry more scientifically and systematically is necessary. For this document to be effectively utilized and realized, more collaboration between various fields is necessary.

Second, the policies lack continuity and stability. The policies are broad, with ambiguous requirements and an absence of a system of sub-goals that align with the overarching plan; there's no differentiation between short-term and long-term objectives. Some documents span 5 years, while others don't specify a timeframe. A significant concern is whether solutions crafted to address current challenges will remain relevant in the future. Enhancing the sustainability of sports industry policies is crucial and challenging, but it can't be achieved immediately and should evolve gradually. Equestrian classes in China remain costly, and reducing their price is an effective method for promoting equestrian sports. Regrettably, no policy or regulation currently addresses this cost concern.

Third, there's a lack of professional assessment and relevance. The horse industry spans several sectors, and each one is distinct. Broad generalizations could hinder the future development of the entire horse industry. Multiple equestrian education systems exist in China, such as the British education system (BHS), the French system (Galop), and the German system (HCCG). Given these varied teaching methodologies, regulating education proves challenging. The CEA riders classification encounters similar issues. Publicly available documents indicate that rider examination and certification is the sole unified component. However, there's no documentation outlining a standardized approach for coach training. Since China lacks a unified equestrian teaching methodology, clubs employ diverse training techniques, complicating oversight. The dearth of technical equestrian experts hampers the growth of the domestic horse industry. From veterinarians to horse handlers, coaches, club managers, judges, and professional equestrian competitors, every facet of equestrian sports faces a shortage of seasoned professionals. This deficiency also restricts the development of equestrian sports. Improve equestrian rules and combine them with China's traits to make a teaching system for equestrian sports that fit in with globalization. Strengthen the assessment so that professional equestrian instructors are licensed to work. Cultivate professional athletes and promote the relationship between universities and enterprises.

Fourth, there's insufficient emphasis on horse welfare. The “National Horse Industry Development Plan (2020–2025)” mentions that an international-standard horse welfare system will eventually be established. This implies that, during budding phase, horse welfare wasn't a priority.

With the growth of equestrianism is tied to the number of clubs, and the proliferation of equestrian clubs relates to land policies (61). Equestrian activities require extensive land areas. When it comes to land-use policies, many city guidelines concerning horse farms are ambiguous. Consequently, several equestrian clubs have encountered issues with unauthorized member elections. Local governments should be in charge of ensuring there is enough land for urban planning and big, functional stadiums. This would help solve the problem of sports for everyone. They should be allocated more to commercial and residential sports land. And should give play to regional advantages, promote regional development, and be suitable for developing regional planning and the formation of regionalized management.

The next focus is on creating disease-free zones. Establishing these zones for equestrian events poses challenges. As per the FEI, a disease-free zone is an area exempt from specified equine diseases within a defined period and under controlled conditions (70). Currently, China has only one such zone in Conghua. However, due to substantial investment requirements, most of its usage has been allocated to the Hong Kong International Jockey Club for training purposes. The quarantine processes for participating horses are intricate, with European horses facing particularly stringent regulations. This has resulted in a limited number of high-profile events in China. The equestrian competition venue for the 2022 Hangzhou Asian Games, set to commence construction in April 2020 in Tonglu, Hangzhou, is anticipated to cost 2.5 billion RMB to create a disease-free zone (71). It's uncertain whether this facility will be sustainably operated and managed post-construction. If the pace of equestrian development accelerates and competition intensifies, maintaining horse health and condition may become feasible. Test the necessity of building epidemic-free zones and plan the follow-up. Considerable investment will be wasted if there is no operation plan for the follow-up site. Rather than constructing it in different zones, we create a single epidemic-free zone and use the same venue for all international equestrian competitions in China.

## Conclusions

China's equestrian industry policies have progressed through essentially three phases: the first phase, from 1978 to 2004; the second, preparation phase, from 2005 to 2012; and the third, development phase, from 2013 to 2022.

If China's equestrianism industry wants to develop sustainably, it should try to solve the following issues. First, there's an issue with the policy's relevancy. The objectives of the policy are vague and difficult to understand, and there's a lack of interaction between the sectors they impact; second, the policy is inconsistent and unstable; third, there's a deficiency in professional assessment and relevance; and fourth, the pace of development is unsustainable.

Overall, the sports management system, the government's policy objectives, and the conflicting interests among policy stakeholders complicate the introduction of changes during policy formulation, implementation, and execution.

## Author contributions

JL: Formal analysis, Methodology, Software, Writing – original draft. RS-G: Project administration, Supervision, Writing – review & editing.

## Footnotes

^1^Mingqi is usually found in Chinese tombs, and it generally refers to burial furniture or objects placed with the intention of providing the deceased with the same physical environment as in life (5).

^2^An international organization that showcases prominent global riders and offers members opportunities in global competitions.
